# Some of the Physiological and Therapeutical Properties of Alcohol

**Published:** 1885-12

**Authors:** J. P. Stevens

**Affiliations:** Macon, Ga.


AMERICAN JOURNAL

OF

DENTAL SCIENCE.

VOL. XIX. THIRD SERIES.—DECEMBER 1885. No. 8.

ARTICLE I.

### SOME OF THE PHYSIOLOGICAL AND THERAPEUTICAL PROPERTIES OF ALCOHOL.

BY J. P. STEVENS, M. D., MACON, GA.

(Read by title at the thirty-sixth Annual Session of the Medical Association of Georgia, at Savannah, Ga., April 15, 1885.)

It is not my purpose to enter upon an elaborate exposition of the subject of my essay, as it would require too extended a monograph for its discussion in its varied relations to society. I will therefore notice only a few points of interest that bear directly upon its employment as a remedial agent in disease.

By the vast majority of physicians the properties of alcohol as a therapeutical agent are regarded as merely a diffusible stimulant without any reference to its function as a nutrient to the tissues and a sustainer of vital energy.

Its medicinal properties, however, are recognized and appreciated by the profession in general.

From the earliest historical times there has not been discovered a race of men who were not in the habit of using as a beverage some form of fermented liquors. "Wine within and oil without" seem to have been regarded as the *summum bonum* for attaining the highest development in the direction of physical and mental activity. It would appear, therefore, that alcohol in some form is a necessity of the animal economy. Wherein consists that necessity?

The protein compounds, albumen, fibrine and casein, form the foundation of tissue development, without which no organic growth can be accomplished. According to Lehman, the blood plasma in a thousand parts shows fibrine 4.05, albumen 78.84, fatty matter 1.72. We see therefore, that nitrogen and its compounds form the basis of organic development, and this is true not only in the animal, but in the vegetable kingdom. Now, we find the chemical analysis of alcohol to be C-4 H-6 O-2. thus showing it to be entirely destitute of nitrogen. How can something come out of nothing is the problem for solution.

Alcohol is produced by the distillation of the products of the fermentation of fruits and the grains of many vegetables. The primary products of fermentation is alcohol and carbonic acid. The further oxidation of alcohol results in *aldehyde* C-4 H-4 O, a highly inflammable volatile liquid, with a pungent, apple-like odor, which passes into acetic acid, and finally into the fermentation of mould.

M. A. Muntz declares that he has discovered traces of alcohol in cultivated soil, rain water, sea and river water and the atmosphere.

Steinmitz says: "I feel compelled to believe, in advance of Leibig, *that alcohol is absolutely generated in the digestive process of all animals.* It is well known that all the vegetables we eat contain starch; all the fruits contain sugar. Now starch can be easily converted into sugar: the process of malting is a familiar instance. . . .

The natural heat of the body is precisely adapted, in the healthy state, to effect a fermentation after having changed the starch into sugar, which last is constantly found in the blood. That alcohol has not been found seems to result chiefly from the fact that it must be sought in arterial blood, or blood which has not lost a portion of its carbon in *transitu*, through the lungs in the respiratory process."

### PROPERTIES OF ALCOHOL.

Alcohol is colorless, highly inflammable, one-fifth lighter than water, a solvent of many substances that are insoluble in water, burns without smoke, and hence is invaluable in the laboratory. It has a powerful attraction for water, which causes it to absorb moisture wherever it can find it; hence its value as an anti-septic. By vaporization it is inhaled into the lungs, diffuses itself through the bronchial tubes, permeates the minute air vesicles and travels with the air vesicles into the circulating current throughout the body. Received into the stomach, it is readily taken by absorption through the veins of the alimentary surface and conveyed by the large venous trunks to the heart. Its strong attraction for water causes it greedily to drink up the moisture of the tissues wherever it comes in contact with them. Hence, the insatiable thirst for water of the inebriate after a fit of debauch. The three main sources of tissue formation and development are the albuminates, the carbo-hydrates and the oleaginous.

The albuminates are concerned in the production of the flesh, blood, bones and nerve. The carbo-hydrates are expended in the evolution of heat and the maintenance of respiration, and the oleaginous in the generation of the fatty tissue. Alcohol being destitute of nitrogen, and having an excess of hydrogen in comparison with its oxygen and carbon, is classed among the carbo-hydrates.

The theory promulgated by Leibig, forty years ago, that the union of the oxygen of the air with the carbon of the blood is the source of animal heat, and the maintenance of the principal that the albuminates alone are concerned in tissue development, would preclude alcohol as a nutritive of the tissues. But this theory has been partially exploded, inasmuch as it is conceded that the carbo-hydrates, although destitute of nitrogen, aid the conversion of the albuminates into tissue. It is well known that in the performance of the organic functions there is a continued retrograde metamorphosis of tissue; and in wasting diseases and long continued starvation, where the equilibrium between supply and waste is not maintained, loss of strength and flesh proceed with commensurate rapidity.

Alcohol, though forming none of the constituents of blood, limits the combination of those constituents, and thus retrograde metamorphosis of tissue is retarded. In moderate quantities, a portion of the alcohol is eliminated through the secretions, and the remainder undergoes combustion for the supply of animal heat. The oxidation necessary for this combustion, without the presence of alcohol, must be expended upon the albumenized tissues to meet the wants of the economy. The amount of alcohol thus utilized by oxidation represents so much tissue saved in the process of retrograde metamorphosis, and therefore so much of conservative energy added to the general fund of force. Alcohol may, therefore, be said to act indirectly as a *nutrient* to the tissues.

Dr. Austie frequently dwells on the notable fact "that in all cases of disease where alcohol is used successfully as a medicinal support, as in the case of exhausting fevers, its presence as an alcoholic emanation, whether in the breath or in the secretions, is absent altogether, as if in those the whole force of the agent was absorbed in its operation."

He also declares "that in snch instances its exciting and intoxicating powers appear to be in abeyance, and that the recovery from acute disease, when this medicine has been successfully employed, is invariably more rapid and complete than it is in altogether simular cases which have been treated without alcohol."

Alcohol may therefore be regarded as a savings bank to the tissues. Drs. Austie, Thudichum and Dupre, in their experiments upon a dog, gave it in ten days two thousand grains of alcohol, and on the last of the ten they administered ninety-five grains of the spirit. Two hours after the last dose the dog was killed, and the whole body, blood, bones, membranes, secretions and brain were subjected to vigorous analysis, and they found but twenty-three and a half grains of alcohol. "The other one thousand nine hundred and seventy-six grains had clearly, therefore, been turned to something else within the living system." Dr. Austie relates the case of an old soldier who for twenty years maintained his bodily structures in good condition with the daily consumption of a pint of gin, and "one small finger length of toasted bread." Another instance is related of a young man reduced by rheumatism, was unable to retain any nutritive substance in his stomach, and for several days was maintained by three-fourths of a pint of gin per day, and made a rapid recovery without any trace of the emaciation and prostration that usually accompany such attacks. The lad, though remarkably sober and steady in his habits while in health, exhibited no indications of intoxication while under treatment, and the abnormal frequency of his pulse and increased respiration gradually recovered their natural standard.

"Dr. D'Lalor also mentions the case of a child only fourteen months old, suffering from inflammation of the lungs, and whose stomach could retain nothing but port wine; for twelve days it subsisted entirely upon wine; it was rapidly cured with no wasting of any account; nor although it drank largely of alcohol, was it ever intoxicated."

May not the therapeutic effects of spirituous liquors, in recovering from the bites of poisonous reptiles, be ascribed to their invigorating and sustaining influence, thus enabling nature to rally her forces and lift up the heavy burden that so fearfully paralyzed her energies until she could accomplish the cure in her own beneficent way?

Dr. Richardson published an elaborate monograph on "Alcohol," wherein he makes this statement: "Alcohol cannot, by any ingenuity of excuse for it, be classed among the foods of man. It neither supplies matter for construction nor heat. It injures construction and it reduces temperature." On the contrary, Brunton avers that alcohol "undergoes combustion in the body; maintaines or increases the body weight and prolongs life on an insufficient diet. It is therefore entitled to be reckoned as a food."

Richardson, prejudiced with a predominant idea, determined to maintain his assumptions at the expense of unfair experimentations. In all his experiments he is said to have administered alcohol in large and toxic doses; hence his deductions will not stand the test of fair philosophical judgment.

How many of our most dangerous mineral and vegetable poisons, when judiciously and carefully employed, are found to be our most valuable therapeutical agents. And here I may remark that there is nothing that so stultifies our intellectual faculties and contracts their operation within the narrow limits of a blind, egotistic self-assertion as prejudice against any line of thought, or the use of any medicinal agent, especially where it may have stood the test of rigid investigation and truthful experimentation.

*Does alcohol lower the temperature of the body?*

Dr. Parks, who has made this query a subject of careful investigations, has arrived at the following conclusions: "In healthy men who have been accustomed to take beer, and sometimes spirits, I could not detect any raising or lowering of the thermometer. Dr. Mainzer found no fall of temperature in trials on himself, but a slight fall in another healthy person. Some experiments by Obenier and Falker are also negative. We may conclude that the effect on temperature in healthy men is extremely slight; there is no increase, and in many persons no decrease. In those in whom there is a slight decrease, the amount is trifling." We may reasonably conclude, therefore, that in moderate quantities the lowering of temperature is comparatively very little. In large and toxic doses it certainly does lower the temperature.

What are the primary effects of a dose of spirituous liquor? We observe, first, an increase in the force and frequency of the heart's action; the pulse becomes fuller, stronger, and more frequent; the vaso-motor nerves of the peripheral blood vessels become partially paralyzed; hence we have injection of the superficial capillaries; a diffused redness pervades the skin, the mental faculties become quickened, and there is a sensation of warmth diffused throughout the body. This determination of the flow of blood from the center to the circumference will be attended with increased radiation of heat from the cuticular surface, and hence it is contended by the alcholo-phobists that the superficial cooling of the blood with its return to the interior of the body will be followed by general reduction in the bodily temperature. But the equilibrium in the circulation is soon restored where the dose of alcohol has not been a large and toxic one, sufficiently so to partially paralyze and greatly weaken the tonicity of the heart's action. When subjected to the influence of severe cold until the body becomes severely chilled, every one will remember how difficult it is to secure a diffused warmth throughout the system even before a hot fire. While one side of the body is being baked the other side continues to be chilled. If, at such a time, even a small portion of spiritous liquor is drunk, it is notable how soon a genial warmth is diffused throughout every part of the body, inducing a degree of equanimity and comfort that is peculiarly grateful. The primary effects of alcohol upon the peripheral capillary system is somewhat analagous to that which we observe in what is called "taking cold." When a draught of cold air flows upon the back part of the neck of a person sitting in a warm room, the primary impression is not induced by the irritation of cold air inhaled through the nostrils, but upon the branches of the sympathetic nerve ramifying upon the scalp. This impression produces a shock to the nerve-centre from which these nerve-fibers proceed. The nerve-centre has connections with other parts of the body, and by reflex action the vaso-motor nerves that control the vessels of the Schneiderian membrane of the nose become partially paralyzed. In consequence these vessels become dilated and engorged, and the shock which produced this congestion continuing, disturbs the equilibrium of the blood supply, and so an inflammatory condition is set up. Nature endeavors to arouse this paralyzed condition of the vessels by the act of sneezing, and thus restore the equlibrium of the circulation. Sometimes she accomplishes her purpose in a few hours, but usually the inflammatory condition is only relieved through the regular stages of recuperation observed in the process of resolution.

If the *modus operandi* of spirituous liquors, as thus considered, be correct, in what diseases are their therapeutical properties especially indicated? Evidently in those of an asthenic type, where vital energy is seriously depressed. In such diseases the retrograde metamorphosis of tissue is usually very rapid. All the excretory organs are weakened in function, and with difficulty they discharge out of the system the debris of broken-down tissue that accumulates in the blood.

In this process of metamorphosis force is lost in the action of chemical force upon the detritus of tissue. Here we have a two-fold loss of energy. If therefore, one of the functions of alcohol is to retard metamorphosis, we can readily perceive its conservative influence in economizing the powers of life, and thus indirectly aiding nature in her recuperative and curative efforts. It is notable that in all fevers the secretion of the gastric and pancreatic glands is kept in abeyance; hence, in exhausting fevers, the feeble powers of assimilation of food, and the usual disgust for food, especially of a nitrogenous kind. Now, as alcohol is classed among the carbo-hydrates, and one of the properties of this class is to aid in the conversion of the albuminates into tissue, it is better to administer stimulants in company with the diet of the patient, whatever we may select. Good fresh milk combines the greatest number of nutritive elements than any other article of diet, and it is usually most digestible. Even in local inflammations and internal congestions of a passive nature, we see no contraindications for alcohol in moderate doses. Its peculiar function of determining the blood to the surface, and thus favoring the maintenance of an equilibrium in the circulation, renders valuable assistance in relieving the internal loaded blood vessels, especially in the latter stages of acute inflammation. In nervous shocks to the system, induced by whatever cause, through tramatic agencies, hysteria, moral impressions, induced by grief or fear, excessive physical or mental exhaustion, there is no agent that so rapidly rallies the sinking energies as vinous and spirituous liquors, not only on account of their rapid absorption and diffusion through the circulation, but they restore warmth to the body, and supply food for the respiratory functions.

In a state of health, during prolonged physical and mental exertion, the sustaining powers of spiritous liquors cannot be relied upon. In all competitive tests, requiring intense and prolonged muscular exertion, tea and coffee, and especially a good, wholesome, nitrogenous diet, which contains all the elements necessary for the regeneration of the wasted tissues, have always been found incomparably superior to alcohol in accomplishing the largest amount of labor. Mr. R. E. Carrington, of Guy's Hospital, in calling attention to the habits of the Cambridge crew when training for competitive prize races, writes: "It is, perhaps, one of the severest tests of muscular power that can possible occur. I find the system pursued to consist of good hours, a moderate amount of good, wholesome food, a moderate amount of stimulants, with plenty of exercise. The stimulants may consist of a pint of beer for dinner, and a similar quantity for supper. A glass or two of good port, or sound claret, are taken during the day. Even champagne is given. Spirits are against all regulations, and are never given, for it is found that they do not tend to strenthen them in any way." It would seem therefore, that a moderate amount of fermented liquors along with food during the day is compatible with the highest development of muscular energy; but that distilled liquors do not increase the bodily strength.

So it is with their influence upon the mental faculties. It is true, that in some temperaments, the highest flights of the poet's fancy and his brighest gems of thought have been reached under the inspiration of the "wine that sparkles in the cup," but it is equally true that profound mental effort that requires severe logical induction is always hindered by the same cause. The brain-cells are stimulated to higher activity, but the thoughts become blurred, ill-defined, run into each other, and are incapable of a clear, incisive analysis. Alcohol, therefore, appears to brighten the imagination, but dulls the preceptive faculties.

It may be well to notice some of the alcoholic preparations employed as medicinal agents. Brandy as obtained, especially from the vintages of the Champagne region of France, containing fifty per cent. of alcohol, is probably the purest of distilled liquors. Gin, of all others, is the most objectionable on account of its adulteration with objectionable substances. Whisky is the most popular and the most extensively used of all distilled liquors, but it is often highly injurious from the presence of fusil oil, which is one of the most pernicious of poisons. The best qualities of wine are without doubt the most wholesome of all alcoholic preparations. It is true that the ordinary wines of commerce are often objectionable from their adulteration with foreign substances, but a good honest quality of dry wine of sufficient age to secure the completion of the fermentative process is, in my judgment, the most suitable for medicinal purposes, especially in exhaustive fevers. It is known that the *torula* plant, concerned in vinous fermentation, continues its work for years after the wine is bottled and all access to atmospheric air is precluded. As a result, various aromatic ethers are evolved which impart the peculiar "bouquet" that renders the wine more exhilarating and acceptable to a weak stomach. On account of the objectionable nature of ordinary commercial wines, we would prefer an honest fermentation of domestic *dry* grape wine, *not less than five years old.* In long continued fevers, it may be given in quantities commensurate with the tolerance of the system and the effects produced. Its good effects will be observed when it slows, and increases the volume of the pulse, and improves the appetite of the patient. Of all wines, sherry and port, are the most objectionable. They are, for the most part manufactured with very pernicious stuffs, such as nitrous ether, sulphate of lime, tannin, alum, and other abominations. Dr. I. Burney Yeo says: "The poor man's ideal of wine is port. But of all the hurtful mixtures that are sold to the poor man, public house port is, perhaps, the worst. I need not enumerate the various subsfances with which it is adulterated, but many of these are astringents, and check the action of the secreting organs." The main idea in selecting a stimulant, whether vinous or spirituous, is to procure one that has completed the process of fermentation, and therefore is sufficiently aged to secure immunity from intermediate products, such as aldehyde and acetic acid. In short, a good, honest article, free from foreign admixtures, and which has been committed entirely to the laboratory of nature to finish her work in her own beneficent way, flavored with aromatized ethers, such as the ancients considered as fit libations to offer to their mythical deities.

It is not our purpose to portray the terrible effects of the immoderate use of alcohol as a social evil. This task belongs to the philanthropist. Alcohol is a double edged sword that often gleams in victorious conflict with disease and death, or cleaves the hopes and fortunes of the wise and virtuous, the innocent and dependent, and consigns to premature destruction millions of our race.—*Atlanta Med. and Surg. Journal.*

